# Depressive symptoms and its associated factors among secondary school adolescents of Birtamod Municipality, Jhapa, Nepal

**DOI:** 10.1371/journal.pgph.0002826

**Published:** 2024-01-19

**Authors:** Rachana Giri, Santosh Khadka, Anisha Chalise, Kshitiz Swar, Shishir Paudel

**Affiliations:** 1 Department of Public Health, CiST College, Pokhara University, Kathmandu, Nepal; 2 Center for Research on Environment, Health and Population Activities (CREHPA), Lalitpur, Nepal; 3 Amity Global Education, Tokha, Kathmandu, Nepal; ICDDRB: International Centre for Diarrhoeal Disease Research Bangladesh, BANGLADESH

## Abstract

Depression is a rising public health concern affecting adolescents’ mental health throughout the world. This cross-sectional study aimed to assess the prevalence of depressive symptoms and its associated factors among adolescents from urban Nepal. The depressive symptoms among 271 randomly selected secondary school adolescents of Britamod Municipality were assessed using Center for Epidemiologic Studies Depression Scale (CES-D). The chi-square test and multivariable logistic regression were executed to assess the statistical relationship between potential risk factors and depressive symptoms at 5% level of significance. The prevalence of depressive symptoms was 42.8% (95% CI: 37.3–49.1%). Multiple logistic regression revealed that female adolescents (aOR: 2.309, 95% CI: 1.233–4.325), adolescents enrolled in higher grades i.e. 10^th^ grade (aOR: 4.576, 95% CI: 1.482–9.128), studying at private school (aOR: 2.302, 95% CI: 1.184–4.476), not sharing their problems with their friends (aOR: 3.022, 95% CI: 1.477–6.186), presence of perceived academic stress (aOR: 2.388, 95% CI: 1.263–4.518), dissatisfaction with current academic performance (aOR: 2.278, 95% CI: 1.141–4.518), lower perceived family support (aOR: 3.440, 95% CI: 1.276–9.275), and lower self-esteem (aOR: 2.139, 95% CI: 1.061–8.708) had higher odds of experiencing depressive symptoms. There is a high prevalence of depression among Nepalese adolescents. The findings highlight the need for health promotion interventions focusing on mental health awareness, enhancing social support systems, and implementing stress reduction strategies within schools to mitigate the burden of depression among Nepalese adolescents.

## Introduction

Depression is a major contributor to the global burden of disease and has impacted a significant number of people across the world [1]. It has become a prevalent form of mental illness, distinguished by symptoms such as sadness, lack of enjoyment, emotions of shame or low self-esteem, disturbed sleep and appetite, feelings of tiredness, and difficulty concentrating [1, 2]. In 2019, approximately 3.8% of the global population, comprising 280 million people, suffered from depression, making it one of the major contributors to the global disease burden [3–5]. The global data also highlights almost 14% of adolescents aged 10–19 years worldwide were affected by any form of mental health conditions in 2021 [6]. Adolescent depression is emerging at an alarming rate throughout the world. A systematic review and meta-analysis revealed that the worldwide pooled prevalence of mental disorders among children and adolescents was 13.4% [7]. In South Asian regions the prevalence of depression among school-going children has been reported at 25.8% in India [8], 17.2% in Pakistan [9], 26.2% in Malaysia [10] and 36% in Sri Lanka [11].

Adolescence is a time of significant changes in the domains of physical, psychological, social, and cognitive development [2, 12]. This period is stressful and may increase their vulnerability to several mental health problems if exposed to violence, abuse, or other stressful events [13]. Depression in adolescence is associated with substantial impairment in functioning and a greater likelihood of experiencing a major depressive episode and other psychiatric disorders in the future [14–16]. It is noteworthy that adolescent depression can impact the path of personality development potentially leading to impaired social functioning in adulthood [17–19]. It can lead to several risk-taking behaviors such as violence, drug and substance abuse, poor dietary habits, alcoholism, and even suicidal ideation among adolescents [20–23]. Thus, by preventing depression, we can protect adolescents from this vicious cycle of maladaptive behavior.

Despite being one of the major public health issues in developing nations, depression is often neglected and Nepal is no exception. According to the National Mental Health Survey of Nepal 2020, the prevalence of mental distress among adolescents is reported to be 5.2% [24]. The past studies focusing on depression among Nepalese adolescents suggest that the overall prevalence of depression among this vulnerable population range from 11.1% to 27% [25, 26]. A past study on depression among higher secondary school adolescents in Nepal by Bhattarai and colleagues [2] suggested a research gap notifying the possible relationship existing between psychosocial school environment and adolescents’ mental health in Nepal but didn’t explore the potential relationship and this study tries to address it. Considering this research gap, this study aimed to assess the prevalence of depressive symptoms and its associated factors among adolescent.

## Methodology

### Study design, population and setting

This was a school based cross-sectional study conducted among randomly selected secondary school students of grade 8, 9 and 10 of Birtamod Municipality from June to September 2022. Britamod Municipality lies in Jhapa District, Koshi Province of Nepal. Based on the estimates from the recent 2021 census, there are a total of 29,852 households, and the area is home to a population of 116,192, with adolescents aged 10–19 years accounting for 21,294 [27].

### Sample size determination

**Fig 1:**
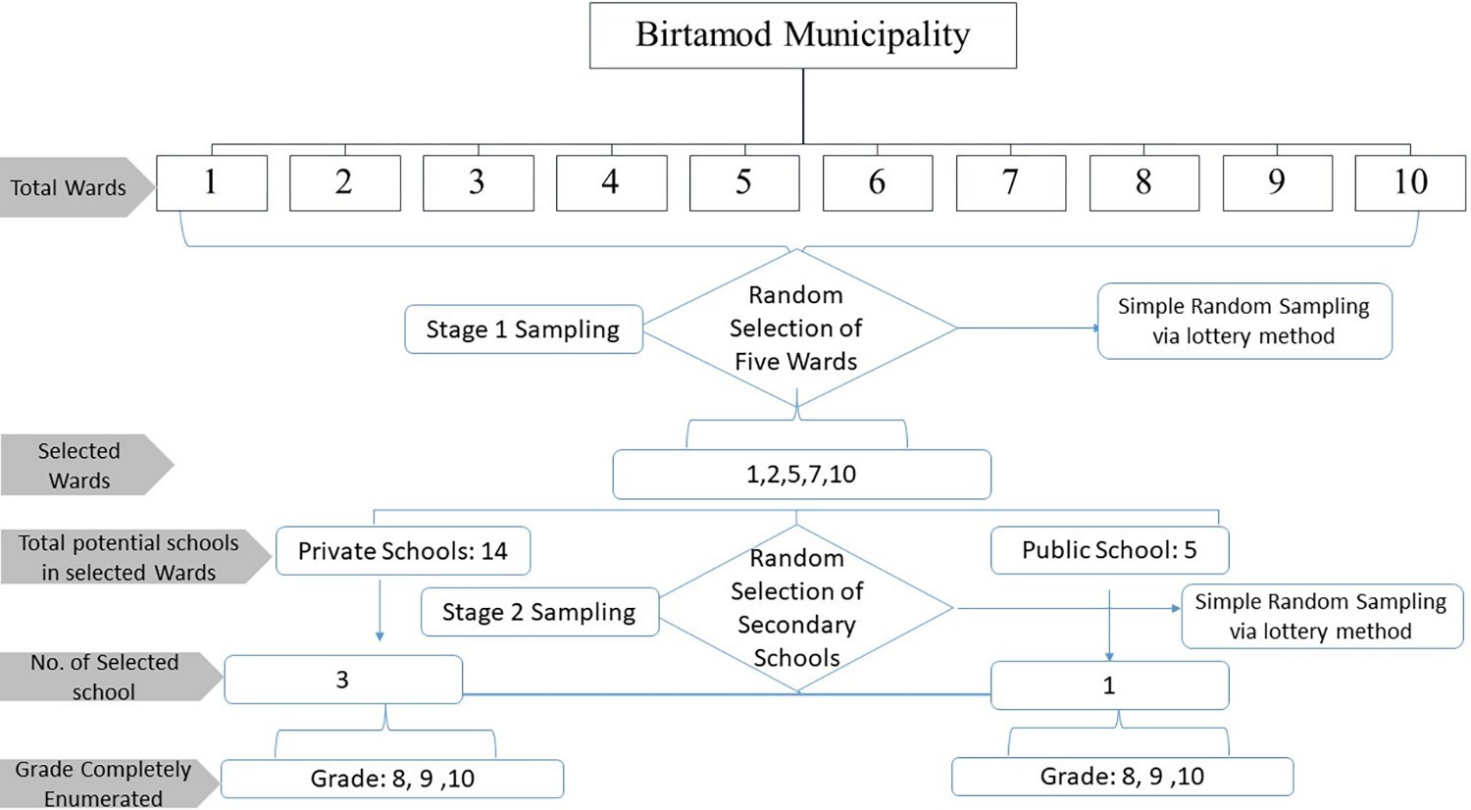
Sampling process.

The sample size for this survey was determined using Cochran’s formula using 13.1% prevalence of depression among adolescent in urban setting of Nepal [28]. Taking this prevalence at 95% confidence interval and 5% margin of error, the initial sample size was calculated to be 175 which was optimized to 290 adjusting the design effect of 1.5 considering multi-stage sampling and non-response rate of 10%. The samples were drawn in multiple stages. Initially, out of a total of ten wards of Birtamod Municipality, five wards were selected at random using lottery method. Then, out of the total public and private schools located in the selected five wards, one public school and three private schools were selected randomly. All the students from grade 8, 9 and 10 of the selected schools were included in the study. The sampling process is illustrated in Fig 1.

### Data collection and study tool

Data was collected using self-administered questionnaire. The school teachers assisted in the data collection by arranging a data collection session for an hour, during which researchers explained the purpose of the survey and distributed the survey questionnaire to the students. The students then completed the survey in their classrooms within the given time frame.

The questionnaire was divided into four sections where, the first section consisted of general information about students’ socio-demographic profile and their academic information such as nature of school, peer and teacher support and their academic performance and self-satisfaction with the performance. Second section consisted of question related to participants’ lifestyle such as physical activeness, internet consumption and sleep pattern and a single question to assess participant’s perceived level of family support. The third section consisted of Rosenberg Self-Esteem Scale [29], translated in Nepali language by Paudel et al. [30] to assess their level of self-esteem. The forth section consisted of Center for Epidemiologic Studies Depression scale (CES-D) [31] to assess the level of depressive symptoms among the adolescents.

The CES-D is a standard tool consisting of 20-items in 4 point Likert scale ranging from 0 (‘rarely or none of the time’) to 3 (‘most or all of the time’) [31]. Higher score in CES-D scale indicates a greater risk of depression. A past study noted that the cut-off of 18 is a better indicator of sub-threshold depression in the Nepali version of CES-D and also revealed a good internal consistency where the Cronbach’s alpha coefficient was noted to be 0.82 [2]. So, the same pre-tested translated version was used in this study and the cut-off of >18 was used to screen depressive symptoms status among the participants.

### Data analysis

For data analysis, all the data was entered in Epi-Data version 3.1 and imported to Statistical Package for the Social Sciences (SPSS) version 22 for analysis. Bivariate analysis was carried out by applying chi-square test to identify the factors associated with depressive symptoms at 95% confidence interval and 5% level of significance. The variables found to be significant in bivariate analysis were subjected to multivariable analysis using binary logistic regression to determine the adjusted effect of each factor on depressive symptoms. Prior to logistic regression, multi-collinearity between the independent variables was tested using variance inflation factor (VIF) test, with a VIF greater than five taken as an indication of multi-collinearity between the independent variables.

### Ethical considerations

This study adheres to the ethical principles outlined in Declaration of Helsinki regarding the use of human subjects in research. Ethical approval for this study was obtained from the Institutional Review Committee of CiST College, Pokhara University (Registration no: IRC/167/078/079). The data was collected only after obtaining necessary permission from academic institutions and written informed consent from all the participants. The parental consent was also obtained from the parents of students who were below the age of 18 prior to their enrollment in the study. To uphold the confidentiality of participant information, identification numbers were assigned instead of using students’ names. No incentives were provided to the participants but a small mental health awareness session was conducted after the data collection session.

## Results

A total of 290 school-going adolescents were approached for data collection, out of which 271 provided complete response to all the questions. Thus, the response rate of 93.45% for all question was acquired and 271 samples were analyzed for this study. Among the 271 participants, 116 reported to have experienced depressive symptoms in past one week based on CES-D score >18, demonstrating the prevalence of depressive symptoms to be at 42.8%. (Table 1).

**Table 1:** Prevalence of depressive symptoms among the adolescents (n = 271).

Depression status	n	% (95% CI)
Depressed	116	42.8 (37.3–49.1)
Non depressed	155	57.2 (50.9–62.7)

Out of 271 participants, nearly half of the participants were females (53.9%). The age of the participants in this study ranged from 12 to 19 years, with a mean age of 13.93±1.50 years. A large majority (77.5%) of the participants reported following Hinduism as their religion. The students from 8^th^ grade comprised of 38.5% of the total participants followed by 32.1% of them from 9^th^ grade and remaining 29.2% from 10^th^ grade. In regards to the academic variables, almost two-third (62.4%) of the adolescents perceived that they experience academic stress. Nearly half (56.1%) of the adolescents reported to have moderate level of perceived family support. More than half (57.9%) of the participants were noted to have moderate self-esteem. In context of their physical activeness, three-fifth (60.1%) of them reported to perceive themselves to have sedentary lifestyle. (Table 2).

**Table 2:** Factors association with depressive symptoms (n = 271).

Characteristics	n (%)	Depressive symptoms		χ^2^	p value
		Present	Absent		
		n (%)	n (%)		
**Age**					
<15 years	171(63.1)	55(47.4)	116(74.8)	21.432	<0.001***
≥15 years	100(36.9)	61(52.6)	39(25.2)		
**Gender**					
Male	125(46.1)	40(34.5)	85(54.8)	11.063	0.001 **
Female	146(53.9)	76(65.5)	70(45.2)		
**Religion**					
Hinduism	210(77.5)	86(41.0)	124(59.0)	2.228	0.526
Buddhism	40(14.8)	21(52.5)	19(47.5)		
Christianity	10(3.7)	5(50.0)	5(50.0)		
Islam	11(4.1)	4(36.4)	7(63.6)		
**Family type**					
Nuclear	188(69.4)	83(71.6)	105(67.7)	0.453	0.501
Joint/ Extended	83(30.7)	33(28.4)	50(32.3)		
**Father’s education**					
Illiterate	16(5.9)	4(25.0)	12(75.0)	4.830	0.305
Literate via informal education	36(13.3)	16(44.4)	20(55.6)		
Primary	40(14.8)	18(45.0)	22(55.0)		
Secondary	145(53.5)	59(40.7)	86(59.3)		
Undergraduate and above	34(12.5)	19(55.9)	15(44.1)		
**Mother’s education**					
Illiterate	25(9.2)	5(20.0)	20(80.0)	9.267	0.055
Literate via informal education	68(25.1)	26(38.2)	42(61.8)		
Primary	56(20.7)	23(41.1)	33(58.9)		
Secondary	101(37.3)	52(51.5)	49(48.5)		
Undergraduate and above	21(7.7)	10(42.8)	11(57.2)		
**Participants Academic Level**					
Grade 8	105(38.7)	24(20.7)	81(52.3)	29.613	<0.001 ***
Grade 9	87(32.1)	44(37.9)	43(27.7)		
Grade 10	79(29.2)	48(41.4)	31(20.0)		
**Type of school**					
Public	109(40.2)	36(31.0)	73(47.1)	7.119	0.008
Private	162(59.8)	80(69.0)	82(52.9)		**
**Perceived academic stress**					
Presence	169(62.4)	85(73.3)	84(54.2)	10.293	0.001**
Absence	102(37.6)	31(26.7)	71(45.8)		
**Satisfaction with academic performance**					
Satisfied	181(66.8)	66(56.9)	115(74.2)	8.949	0.003**
Unsatisfied	90(33.2)	50(43.1)	40(25.8)		
**Perceived family support**					
Low	39(14.4)	21(18.1)	18(11.6)	13.044	0.001**
Moderate	152(56.1)	74(63.8)	78(50.3)		
High	80(29.5)	21(18.1)	59(38.1)		
**Problem sharing with friends**					
Present	205(75.6)	76(65.5)	129(83.2)	11.293	0.001**
Absent	66(24.4)	40(34.5)	26(16.8)		
**Self-esteem**					
Low	21(7.7)	12(10.3)	9(5.8)	8.476	0.014*
Moderate	157(57.9)	75(64.7)	82(52.9)		
High	93(34.3)	29(25.0)	64(41.3)		
**Perceived physical activeness**					
Active	108(39.9)	43(37.1)	65(41.9)	0.656	0.418
Sedentary	163(60.1)	73(62.9)	90(58.1)		
**Daily Internet consumption**					
< 4 hours	249(91.9)	105(42.2)	144(57.8)	0.506	0.477
≥4 hours	22(8.1)	11(50.0)	11(50.0)		
**Average sleep duration**					
<5 hours	31(11.4)	14(45.2)	17(54.8)	0.135	0.935
5–7 hours	176(64.9)	74(42.0)	102(58.0)		
≥7 hours	64(23.6)	28(43.8)	36(56.3)		

*Statistical significance at p<0.05

**Statistical significance at p<0.01

***Statistical significance at p<0.001

The bivariate analysis performed through χ^2^ test revealed the socio-demographic factors such as age and gender to have a statistically significant relationship with adolescent depressive symptoms at p<0.05. However, participant’s religion, family type, and parental education were not associated with depressive symptoms. A statistically significant relationship existed between adolescents’ depressive symptoms and their academic attributes such as academic level, type of school, perceived academic stress and satisfaction with academic performance. Similarly, adolescents’ level of perceived family support, problem sharing behavior, and level of self-esteem were also found to be associated with depressive symptoms. (Table 2).

For multivariate analysis, the VIF test was performed among independent variables found to have statistically significant relationship with depression in bivariate analysis, where the highest reported VIF was 1.840, indicating that there was no issue of multicollinearity. It was observed that female adolescents were twice (aOR: 2.309, 95%CI 1.233–4.325) more at odds of experiencing depression than male adolescents. Similarly, in comparison to adolescents enrolled in 8^th^ grade, there was two-folds increase in odds of depression among adolescents enrolled in 9^th^ grade (aOR: 2.420, 95%CI 1.091–5.365) and four-folds increase among adolescents enrolled in 10^th^ grade (aOR: 4.576, 95%CI 1.482–9.128). Likewise, students studying in private school were twice (aOR: 2.302, 95%CI 1.184–4.476) more at odds of experiencing depression as compared to adolescents studying in public schools. Similarly, adolescents not sharing problems with their friends were thrice (aOR: 3.022, 95%CI 1.477 to 6.186) more at odds of experiencing depression as compared to those who shared their problems with their friends. Also, it was seen that students experiencing academic stress had two-folds (aOR: 2.388, 95%CI 1.263–4.518) increase in odds of depression than who didn’t. Likewise, students experiencing dissatisfaction with current academic performance were twice (aOR: 2.278, 95%CI 1.141–4.518) more at odds of experiencing depression. Similarly, students having lower perceived family support were thrice (aOR: 3.440, 95%CI 1.276–9.275) more at odds of facing depression than who had higher perceived family support. Similarly, in comparison to adolescents having higher self-esteem, there was two-folds increase in odds of depressive symptoms among adolescents having moderate self-esteem (aOR: 2.009, 95% CI 1.017–3.969) and two-folds increase among adolescents having lower self-esteem (aOR: 2.139, 95%CI 1.061–8.708). (Table 3).

**Table 3:** Predictors of depressive symptoms among school adolescents.

Characteristics	UOR (95% CI)	p-value	AOR (95% CI)^#^	p-value
**Age**				
<15 years	Ref		Ref	
≥15 years	3.299 (1.973–5.517)	<0.001*	2.109 (0.945–5.264)	0.110
**Gender**				
Male	Ref		Ref	
Female	2.307 (1.404–3.791)	0.001*	2.309 (1.233–4.325)	0.009*
**Participants Academic Level**				
Grade 8	Ref		Ref	
Grade 9	3.453(1.858–6.418)	<0.001*	2.420 (1.091–5.365)	0.030*
Grade 10	5.266 (2.752–8.925)	<0.001*	4.576 (1.482–9.128)	0.008*
**Type of school**				
Public	Ref		Ref	
Private	1.978 (1.195–3.275)	0.008*	2.302 (1.184–4.476)	0.014*
**Problem sharing with friends**				
Present	Ref		Ref	
Absent	2.611 (1.478–4.614)	0.001*	3.022 (1.477–6.186)	0.002*
**Perceived academic stress**				
Presence	2.318 (1.380–3.893)	0.001*	2.388 (1.263–4.518)	0.007*
Absence	Ref		Ref	
**Satisfaction with academic performance**				
Satisfied	Ref		Ref	
Unsatisfied	2.178 (1.302–3.643)	0.003*	2.278 (1.141–4.518)	0.020*
**Perceived family support**				
Low	3.278 (1.489–7.315)	0.004*	3.440 (1.276–9.275)	0.015*
Moderate	2.669 (1.476–4.813)	0.001*	3.774 (1.825–7.807)	0.001*
High	Ref		Ref	
**Self-esteem**				
Low	2.943 (1.116–7.756)	0.029*	2.139 (1.061–8.708)	0.035*
Moderate	2.019 (1.177–3.460)	0.011*	2.009 (1.017–3.969)	0.044*
High			Ref	

^#^Logistic regression model adjusted for all variables in the table, Nagelkerke R^2^ = 0.410, Hosmer-Lemeshow χ^2^ = 8.066, p = 0.427

*Statistical significance at p<0.05; AOR: adjusted odds ratio; UOR: unadjusted odds ratio.

## Discussions

The prevalence of depressive symptoms among adolescents studying in secondary school of Birtamod Municipality was found to be 42.8% which is near to the prevalence observed among higher secondary school adolescents of Pokhara Metropolitan city at 44.2%, using the CES-D tool for screening [2]. However, the current prevalence is slightly higher than the prevalence noted among secondary school adolescents of Kathmandu Metropolitan city, which was at 25.9% [32]. The prevalence of mental disorder among Nepalese adolescents of age 13–17 years was noted to be at 5.2% by the National Mental Health Survey of Nepal of 2020 [33]. Although the national average seems to be low, other cross-sectional studies conducted in different part of the country suggest that overall prevalence of depression among adolescents lies between 11.1% and 56.5% [25, 26, 28, 34, 35]. This difference in rate of depressive symptoms observed among Nepalese adolescents might be due to the difference in nature of the study area and the diverse social and ethnic characteristics of the country. This high prevalence of depressive symptoms highlights the raising concern of mental wellbeing among Nepalese adolescents.

The studies based on Southeast Asian (SEA) region also suggest an alarming rate of depression among the adolescents as 34.9% of the adolescents were reported to experience depression in Thailand [36], 33.1% in Malaysia [37], 29.3% in Indonesia [38] and 27.2% in Myanmar [39]. A study conducted among higher secondary school students of North Kerala, India reported that clinically significant depression lies as high as 57.7% [40]. This reveals that there is a high prevalence of depressive symptoms among the adolescents which is a raising public health concern.

In this study, the socio-demographic factors such as religion, family type, mother’s education were not found to have statistical association with depressive symptoms. These findings are in line with the findings shared by the study based on secondary level students of Kathmandu Metropolitan, observing no statistical relationship between these socio-demographic factors and depression [32]. Similar to the finding of this study, another study from Nepal revealed that female adolescents were almost twice more at odds of being depressed than their male counterparts [35]. Similar phenomenon has also been observed in other nations such as Malaysia and Central Uganda, where female were found to have almost two-to-three-folds increase in odds of depression than males [41, 42]. Female adolescents experience hormonal changes and stressful events during puberty much more than males, which might make them more prone to anxiety and stress [34]. The hormones such as estrogen and progesterone have been noted to affect different regions of brain such as hippocampus, thalamus and brain stem which are responsible for modulation of mood and behavior [43]. The fluctuation in these hormones has also been linked to premenstrual syndrome (PMS) and premenstrual dysphoric disorder (PMDD) [44]. All of these might be the reason for the females adolescents to be more vulnerable to depressive symptoms than males.

This study noted that school environment and academic factors can have a major influence on adolescent’s mental health. The students enrolled at private schools were found to have higher odds of being depressed than those from public school. Similarly, higher the academic level of the students, higher was their odds of experiencing depressive symptoms. Similar observation was also shared by a past study from Northern India where private school students were found to have three-fold increase in odds of depression as compared to public school students [45]. In contradiction, another study from India noted the prevalence of depression and anxiety to be higher among adolescents of public schools, whereas the level of stress was observed to be higher among the private school adolescents [46]. Likewise, another study based on school going adolescent of India also noted that higher grade students were 1.6 times more likely to be depressed than students at lower grades [47]. The reason for the students from private schools and higher grades to be more depressed might be linked with higher level of stress, academic pressure and competitive environment.

It was observed that presence of perceived academic stress is one of the important factors linked with depressive symptoms. This is in line with the findings shared by a study based on urban Nepal where students who perceived higher academic stress were reported to have higher likelihood of experiencing mental distress [34]. Likewise, it was also observed that students who were not satisfied with their academic performance were more likely to be depressed than the students who were satisfied. In line with this study, another study from rural Nepal noted that those students who were not satisfied with academic performance were twice more likely to be depressed than those who were satisfied [26]. The academic stress and dissatisfaction with one’s own performance might lead the adolescents through feeling of inadequacy and overwhelming pressure to perform better, and might lower their confidence and self-esteem.

The psychosocial factors such as lower level of perceived family support, not sharing their problems with peers as well as lower level of self-esteem among the school adolescents were found to have a significant relationship with depressive symptoms. Another study from Nepal revealed the interconnection between perceived level of social support and individual’s self-esteem, suggesting that perceived social support could be affecting the mental health by affecting individual’s self-esteem [30, 48, 49]. It was also suggested that family members can be the primary factor influencing the person’s self-worth leading further towards higher self-esteem [30]. Similar observations were also made in other nations as Ethiopian adolescents with lower level of social support were found to be six times more likely to develop depression than those who had moderate level of social support [50]. Similarly, the Vietnamese students with lower self-esteem were nearly five times more likely to experience depression than those with higher self-esteem [51]. The negative correlation between mental distress and self-esteem has been well established among students group [30]. These findings suggest that since social support and self-esteem contributes significantly to the mental wellbeing of the students, screening for poor support system and low self-esteem can possibly be a good strategy to identify student’s risk of mental health problems as well as other social and behavioral problems.

### Limitations

While our study is among the few studies that have assessed the depressive symptoms and its statistical relationship with academic and social factors among school adolescents in context of Nepal, it is crucial to acknowledge its inherent limitations, and the findings should be interpreted based on these limitations. Despite our efforts to mitigate reporting biases in this self-reporting survey, which may arise from factors such as social desirability, recall bias, or varying interpretations of symptoms in the survey questions, through the provision of a private space at the school during data collection and conducting an orientation session to clarify each survey question, it is important to acknowledge that these challenges may still persist. Although we tried to cover the school adolescents from both public and private sectors of Birtamod Municipality, Nepal being a small yet culturally rich and diverse country, the study area still might not have captured all the cultural, racial and ethnic diversity of the country. Moreover, given its institution-based nature, the study exclusively addresses school adolescents, potentially underrepresenting the prevalence of depressive symptoms among adolescents within community settings. Thus, a larger community-based survey covering these factors might provide additional insights over mental health issues and challenges among Nepalese adolescents.

### Conclusion

This study highlights the pressing issue of escalating depressive symptoms rates among Nepalese adolescents. Females, adolescents enrolled at private schools, those studying in higher academic grades, experiencing academic stress, having poor perceived social support and having lower self-esteem were found to be significantly associated with depressive symptoms. Thus, there is a need for targeted interventions to enhance mental health awareness, provide gender-sensitive counselling support and foster positive school environment to manage academic stress, and psychosocial well-being through improved family support and self-esteem-building programs for adolescents in Nepal.

## Supporting information

S1 Data Data used in this study. (SAV)
